# Development of a DNA Aptamer for Screening *Neisseria meningitidis* Serogroup B by Cell SELEX

**DOI:** 10.22034/ibj.22.3.193

**Published:** 2018-05

**Authors:** Kimia Mirzakhani, Seyed Latif Mousavi Gargari, Iraj Rasooli, Samaneh Rasoulinejad

**Affiliations:** 1 Faculty of Medicine, Institute of Human Genetics, Friedrich-Schiller University, Jena, Germany; 2 Department of Biology, Shahed University, Tehran, Iran; 3 Molecular Microbiology Research Center, Shahed University, Tehran, Iran; 4 Max Planck Institute for Polymer Research, Mainz, Germany

**Keywords:** Aptamer, Flow cytometry, *Neisseria meningitidis*, Serogroup

## Abstract

**Background:**

Artificial oligonucleotides like DNA or RNA aptamers can be used as biodiagnostic alternatives for antibodies to detect pathogens. Comparing to antibodies, artificial oligonucleotides are produced easily at lower costs and are more stable. *Neisseria meningitidis*, the causative agent of meningitis, is responsible for about 1% of infections in an epidemic period. Specific DNA aptamers that bind to *N. meningitidis* serogroup B were identified by whole-cell Systemic Evolution of Ligands by EXponential Enrichment (SELEX).

**Methods:**

The SELEX begins with a library of labeled ssDNA molecules. After six rounds of selection and two rounds of counter-selection, 60 clones were obtained, of which the binding efficiency of 21 aptamers to the aforementioned bacterium was tested by flow cytometry.

**Results:**

The aptamers K3 and K4 showed the highest affinity to *N. meningitidis* serogroup B and no affinity to *N. meningitidis* serogroups Y, A, and C, or to other meningitis causing bacteria. The dissociation constant (K_d_ value) for K3 and K4 were calculated as 28.3 ± 8.9 pM and 39.1 ± 8.6 pM, respectively. K3 aptamer with the lowest K_d_ was chosen as the main aptamer. K3 could detect *N. meningitidis* in patients’ cerebrospinal fluid (CSF) samples and in CSF from healthy volunteers inoculated with *N. meningitidis* serogroup B (ATCC 13090) at 200 and 100 CFU ml^-1^, respectively.

**Conclusion:**

The findings suggest the application of the developed aptamer in specific detection of *N. meningitidis* serogroup B amongst a group of meningitis causing bacteria.

## INTRODUCTION

Despite advances in controlling infectious diseases, *N. meningitidis* has been life-threatening to human populations for more than twenty decades[1]. Invasive meningococcal disease represents a serious, but potentially preventable, healthcare problem in both developed and developing countries. The onset of this frequently life-threatening disease is rapid, and characteristic symptoms emerge relatively late, making the infection difficult to diagnose[2].

Mortality even with appropriate antibiotic treatment remains high at 10-15%[3]. *N. meningitidis* adaptation for survival in the human nasopharynx of about 10% of the population makes the meningococcus a highly successful commensal bacterium, and sometimes a fulminant and fatal pathogen represents an important case study in microbial pathogenesis[4,5].

*N. meningitidis* is surrounded by a complex polysaccharide capsule that is essential for its pathogenesis[6]. Based on the chemical composition of the capsular polysaccharide, the bacterial species is further categorized into 13 serotypes with five serotypes viz., A, B, C, W-135, and Y, being the most clinically relevant, causing the overwhelming majority of diseases[7] with the serotype B absent in Africa[8]. So far, no single vaccine is available against all serogroup B meningococcal strains. Epitopes are found in their capsular polysaccharide cross- or non-cross-reacting with human polysialic acid[9]. *N. meningitidis* is a systemic pathogen showing large bacterial aggregates in a close association with the vascular wall of small vessels. The ability of this bacterium to colonize blood vessel endothelium is likely to impact its capacity to both multiply in the blood stream and to reach the brain[10]. Meningococcus can cross the blood-brain barrier leading to meningitis[11]. Colonization rates increase after infancy, reaching a peak of 10-35% in early adulthood, but thereafter decline to less than 10% in older age groups[12], although this could be an underestimate[13].

Detection and identification of bacterial species are major issues for clinical diagnosis[14]. Since meningitis infections has high rate of severity, a quick detection and identification of *N. meningitidis* are critical. *Neisseria* cells isolation from cerebrospinal fluid (CSF) is difficult as a microaerophilic environment with specific thermal requirements is necessary. Confirming *Neisseria* in CSF is time-consuming that may take 2-3 days[15]. The polymerase chain reaction, as a diagnostic tool, is based on priming specific DNA sequences of interest and amplifying them. *N. meningitidis* gene-specific primers have been designed and used in genogrouping of this bacterim[16]. However, due to the highly variable nature of the organism’s genome, it is difficult to choose a gene conserved in any *N. meningitidis* with a specific sequence only to the species of interest and not to other bacteria[17]. Therefore, rapid and sensitive detection methods for *N. meningitidis* are important. Biosensors are of great potential in this respect, and aptamers are attractive tools with regards to their characteristics such as their specificity, small size, easy synthesis, absence of immunogenic reactions, and lower expenses[18-20].

Aptamers are powerful capturing probes against various targets such as proteins, small organic compounds, metal ions, and even cells[21,22]. Numerous reports have detailed the selection of aptamers against different bacterial species[23-28]. In cell-SELEX, the whole of the cell has been shown to be targeted[20,22,29]. Sheikhzadeh *et al*.[30] used copolymer [pyrrole-co-3-carboxyl-pyrrol]-conjugated aptamer to develop a label-free electrochemical biosensor for the detection of *Salmonella typhimurium*. We have previously selected aptamers for recognition of *Hemophilus influenza*, group A *Streptococcus* serotype M3, and *Acinetobacter baumannii* isolates[31-33]. Here, we report the development of an aptamer that binds with high affinity and specificity to *N. meningitidis* serogroup B. To our knowledge, no report of aptamers targeting various *N. meningitidis* is available. This report is the first on specific pre-labeled fluorescent DNA aptamers on *N. meningitidis*.

## MATERIALS AND METHODS

### Bacterial strains, culture conditions, and apparatus

All bacterial strains were from the American Type Culture Collection (ATCC), including *N. meningitidis* serogroup B ATCC 13090, as the target, as well as *Neisseria lactamica* ATCC 23970, *Listeria monocytogenes* ATCC 7644, *Escherichia coli* O1:K1:H7 ATCC 11775, *Staphylococcus aureus* ATCC 25923, *Acinetobacter baumannii* 19606, *Hemophilus influenzae* type b (*Hib*) ATCC 10211, *Streptococcus pneumonia* ATCC 33400, as counter targets. *N. meningitidis* serogroups Y ATCC 35561, A ATCC 13070, and C ATCC 13102 were used to compare the serogroups affinity. 

All bacterial strains were grown in Brain Heart Infusion broth (except *N. meningitidis* and *Hib* that were grown in chocolate agar) at 37 °C overnight and harvested in log-phase growth. For Systemic Evolution of Ligands by EXponential Enrichment (SELEX), *N. meningitidis* cells from 10-ml overnight broth cultures were washed three times in 1× PBS (8.0 g NaCl, 0.2 g KCl, 1.44 g Na_2_HPO_4_, 0.24 g KH_2_PO_4_ in 1 L, pH 7.4) and finally suspended in 500 μl PBS. A 10-μl volume of this solution (approximately 10^7^-10^8^CFU ml^-1^) was used for each round of SELEX. For counter-SELEX, 2 ml aliquots of overnight cultures of other bacteria were pooled and washed in PBS, and the pellet resuspended in 1 ml PBS. A volume of 100 μl of this solution was used in the counter-SELEX. *E. coli* TOP10 cells (Invitrogen, USA) were used for all transformations. The initial single-stranded DNA (ssDNA) library and primers were obtained from Metabion (Germany) and TAG Copenhagen A/S (Denmark), respectively. Lambda exonuclease (1000 Units) was purchased from Thermo Fisher Scientific Company (USA) and BSA from Sisco Research Laboratories Pvt. Ltd. (SRL, India). Fluorescent detection assays were performed using Sysmex Partec GmbH (Germany).

### Preparation of DNA library and aptamer selection

The library sequences, forward primer, and fluorescent labels forward primer, reverse primer, and phosphate reverse primer are shown in Table 1. The diluted aptamer library (10 μM initial concentration) was amplified in 50-μl PCR reactions using a three-step thermal protocol consisting of initial denaturation at 95 °C for 5 min, followed by 30-35 cycles of 95 °C for 1 min, 58-62 °C for 1 min, 72 °C for 1 min, and a final extension at 72 °C for 5 min. The SELEX was initiated with the ssDNA library (2 nmol) denatured by λ exonuclease, a highly processive 5′→3′ exonuclease that degrades double-stranded DNA[34]. Five µg of the purified dsDNA was incubated with 5U lambda exonuclease in a total reaction volume of 30 µL in 1× lambda exonuclease reaction buffer at 37 °C. The reaction was terminated by 10-min incubation at 80 °C and flash cooled on ice for 12 minutes after 0, 15, 25, 35, 45, and 60 minutes of digestion. ssDNA was incubated in 250 μl of 1× binding buffer (1× PBS, 0.05% BSA and 0.02% Tween 20) at 37 °C for 1 h, together with 10^8^ CFU/ml of target cells with gentle shaking on a rotary shaker. The ssDNA-bound cells were recovered by centrifugation at 6000 ×g at 4 °C for 10 min and washed to remove unbound and non-specifically bound ssDNA moieties. The precipitant was then diluted by adding 100 μl of 1× PCR reaction buffer, boiled for 8 min, snap cooled on ice for 5 min, and extracted by centrifugation as described above. The supernatant was used as PCR template to obtain the ssDNA pool for the next round of selection.

**Table 1:** Oligonucleotides used in the selection and characterization of aptamers with binding affinity to *N. meningitidis* serogroup B

Name	Oligonucleotides
DNA aptamer library	5′-GCCTGTTGTGAGCCTCCTAAC(N38)CATGCTTATTCTTGTCTCC-3′
Forward constant region primer	5′-GCCTGTTGTGAGCCTCCTAAC-3′
FAM-forward constant region primer	5′-FITC-GCCTGTTGTGAGCCTCCTAAC 3′
Reverse constant region primer	5′-GGGAGACAAGAATAAGCA-3′
P-reverse constant region primer	5′-phosphate-GGGAGACAAGAATAAGCA 3′

The counter-selection against a mixture of other related meningitis bacteria, including *N. lactamica*, *L. monocytogenes*, *E. coli* O1:K1:H7, *S. aureus*, *A. baumannii*, *H. influenza* type b, *S. pneumoniae*, and *N. meningitidis* type Y were introduced in the 3^rd^ and the 5^th^ rounds in order to ensure species specificity of aptamers to the target bacterium. A total of six rounds of SELEX and two rounds of counter-SELEX were performed to select specifically high-binding aptamers toward *N. meningitidis*. In order to improve the affinity and specificity of binders, the amount of input ssDNA was reduced (from 2 nmol to 100 pmol) at each round of selection along with descending the incubation time from 45 min to 15 min. To monitor the enrichment of aptamers in the selected ssDNA aptamer pool, the outcome of SELEXs 3^rd^, 7^th^, and 8^th^ were amplified with fluorescence isothiocyanate-labeled primer and evaluated with flowcytometry. The enriched ssDNA aptamer pool of the last SELEX was PCR amplified, and the products were cloned into the pTG19-T vector, which was then transformed into *E. coli* TOP10 cells. The colonies resulting from the transformation were tested by colony PCR using simple primers to isolate the positive clones.

### Binding characterization of aptamers by fluorescence-activated cell sorting (FACS)

In order to monitor the enrichment of aptamers in the selected ssDNA pool, *N. meningitidis* was prepared with 1.0×10^8^ CFU ml^-1^ in 100 μl PBS. This analysis was used to assess the binding of the individual selected ssDNA to Neisseria cells. The selected DNA was fluorescently labeled via PCR amplification with 5′-FAM. The dsDNA was denatured to ssDNA by λ exonuclease. Binding assays were then carried out by incubating 50 pM of fluorescently-labeled aptamer candidates with 10^8^ cells in PBS for 45 min and then by a single washing of the cells in washing buffer (1× PBS and 0.02% Tween 20). The cells were then resuspended in 100 μl PBS for flow cytometric analysis. This method called FACS analysis and was used for measuring cell-assorted fluorescence[35]. The affinity of the aptamer candidates to each serogroup was assayed. Fifty picomolar (pM) of fluorescently-labeled aptamer was mixed with 10^8^ CFU/ml *N. meningitidis* cells suspended in 250 μL binding buffer and incubated at 4 °C for 45 min. The mixture was then washed and resuspended in 100 μL PBS for flow cytometry analysis. In addition, bacteria cocktail used for counter-SELEX was also tested for cross-reactivity using FITC-labeled aptamer candidate.

### Estimation of dissociation constant

The equilibrium dissociation constant (K_d_) of each selected aptamer was measured by performing binding assays with overnight grown *N. meningitidis* cells (10^7^~10^8^ CFU ml^-1^) using different concentrations of the aptamer (i.e. 0, 10, 50, 100, 200, and 300 pmol). In brief, *N. meningitidis* cells were bound with various concentrations of aptamers and washed three times, and the cell suspension was treated with 50 pM of FITC tags prior to subjecting to flow cytometry (n=20,000). The equilibrium dissociation constant (K_d_) was calculated by plotting the average total percentage of fluorescent bacterial cells (Y), which corresponded to aptamer-bound *N. meningitidis*, against the concentration of aptamer (X)[36]. SigmaPlot 12.0 software was used to fit a nonlinear regression curve from which the *K*_d_ values were estimated.

### Structural prediction

The structural folding (secondary structure) of the select aptamer sequences was predicted using the online software OligoAnalyzer 3.1 (https://www.idtdna.com/calc/analyzer). The modeling was done assuming ionic conditions of 144 mM Na^+^ at 21 °C.

### Detection of N. meningitidis cells in CSF

#### Natural infection

CSF samples, collected from *N. meningitidis*-infected patients who admitted in Sina Hospital, Tehran, Iran, were diluted to 200 CFU ml^-1^ *N. meningitidis*. The samples were treated with 50 pM of FITC-labeled aptamer. They were further diluted to 100 CFU ml^-1^ to examine the sensitivity of the aptamer. The CSF sample from a healthy individual served as the negative control.

#### Artificial infection

The sterile CSF from healthy volunteers was inoculated with *N. meningitidis* serogroups B (ATCC 13090) and another serotype (ATCC 35561) at 10^2^ and 10^8^ CFU ml^-1^ concentrations in order to rule out the interfering role of any other factor present in CSF[31].

## RESULTS

### SELEX optimization and FACS

**Fig. 1:**
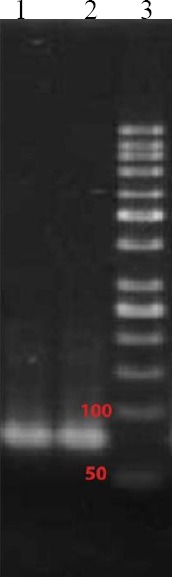
PCR-amplified nucleic acid fractions of initial DNA library. Lane 1 and 2, amplified DNA library; lane 3, DNA ladder (50 bp).

The reaction of λ exonuclease was terminated at 25^th^ minute after digestion where ssDNA was obtained. The single-band intensity was decreased with time course; therefore, digestion time of 25 minutes was chosen as the optimum time. Maximum PCR product was at 62 °C as a result of which a single 78-bp band was noted (Fig. 1). After the 8^th^ round of selection, the evolution of the ssDNA pool was stopped, and selected ssDNAs were amplified with unmodified primer sets. The aptamer pools transformants were cloned, resulting in a total of 60 unique aptamers obtained from the SELEX process of which 21 were selected for flow cytometry, binding affinity (n=20,000). After analysis of these transformants, 8 unique aptamers viz., 1, 2, 3, 4, 5, 31, 39, and 40 with 0.82, 0.5, 2.74, 0.73, 0.79, 0.5, 0.3, and 1.14% affinity, respectively were chosen. The binding interaction of the aptamers to *N. meningitidis* cells (10^7^-10^8^) was screened using 50 pM of each sequence.

### Affinity of the selected aptamers and cross-SELEX

**Fig. 2:**
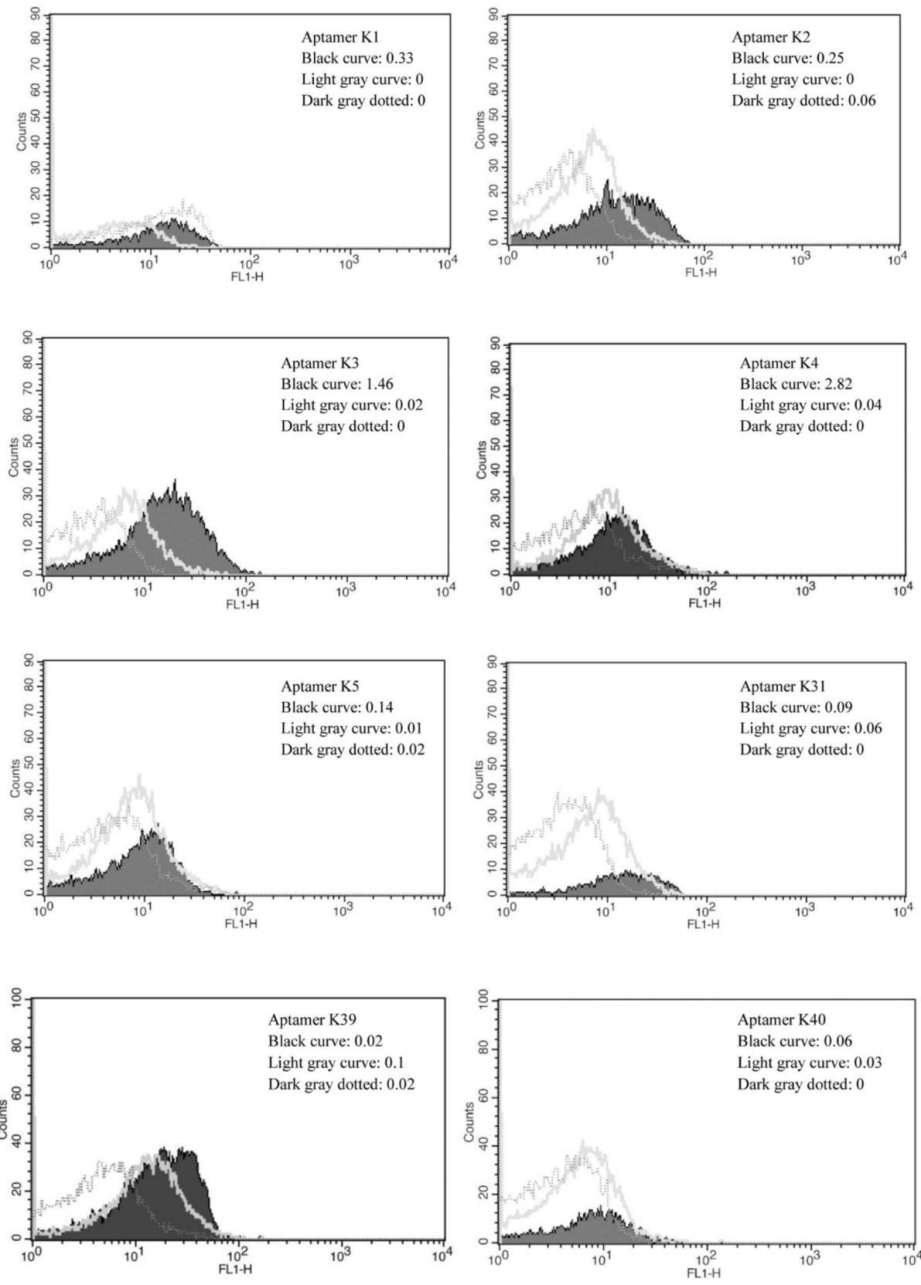
The fluorescent affinity of aptamers K1, 2, 3, 4, 5, 31, 39, and 40 to *N. meningitidis* serogroup B (black curve), *N. meningitidis* serogroup Y (light gray curve), and cross-SELEX (dark gray dotted).

Eight unique aptamers were chosen to compare the fluorescent intensity between *N. meningitidis* serogroups and several other bacteria causing meningitis. Higher percentage was found in K3 and K4 sequences (1.46 and 2.82%) of fluorescence to *N. meningitidis* serogroup B as compared to 0.02 and 0.04% to *N. meningitidis* serogroup Y and no affinity to counter bacteria in Cross-SELEX (Fig. 2). Due to the high binding affinity of K3 and K4, these aptamers were chosen for further characterizations.

### Dissociation constants

**Fig. 3:**
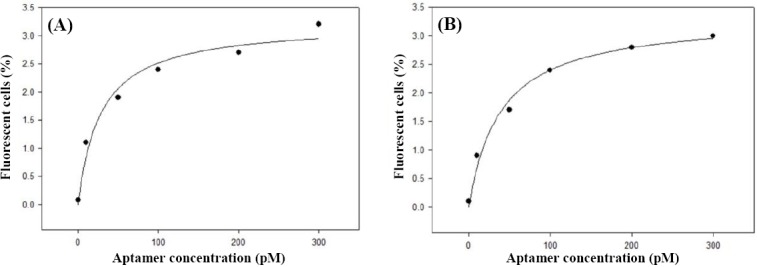
The equilibrium dissociation constant (K_d_) of aptamers (A) K3 and (B) K4

The binding efficiency of the aptamers K3 and K4 to *N. meningitidis* serogroup B cells was evaluated within aptamer concentration range of 0-300 pM and a constant cells population of 10^8^ CFU/ml for each assay. Aptamer K3 bound 0.08, 1.1, 1.9, 2.4, 2.7, and 3.2% cells at 0, 10, 50, 100, 200, and 300 pM of aptamer concentrations, respectively, and binding affinities for K4 at the same concentrations were 0.1, 0.9, 1.7, 2.4, 2.8, and 3% respectively. The K_d_ value for aptamer K3 was 28.3 ± 8.9 pM, and that of K4 was 39.1 ± 8.6 pM (Fig. 3).

### Binding affinity of aptamer K3 to N. meningitidis serogroup B

**Fig. 4:**
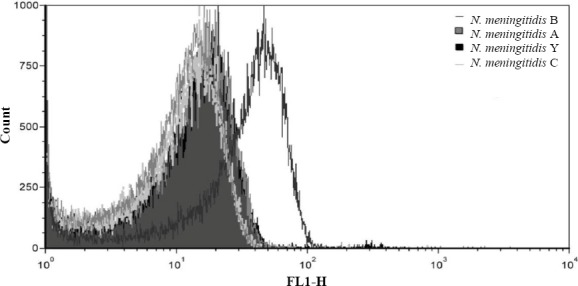
Binding affinity of aptamer K3 to *N. meningitidis* serogroup B comparing to other serogeoups. Binding analysis of aptamer (K3) apparent binding of 0.03%, 0.21%, and 0 was observed for *N. meningitidis* serogroups Y, A and C cells, respectively, whereas that of *N. meningitidis* serogroup B was as high as 2.78%.

On the basis of K_d_ value for aptamers K3 and K4, aptamer K3 was selected for further characterization. Similar to the process mentioned above, *N. meningitidis* serogroups B, Y, A, and C (1.0×10^8^ CFU/ml) were titrated against aptamer K3 and analyzed by flow cytometry. Aptamer K3 had the highest affinity to *N. meningitidis* serogroup B as compared to *N. meningitidis* serogroups Y, A, and C (Fig. 4).

### Prediction of unique secondary structure

**Fig. 5:**
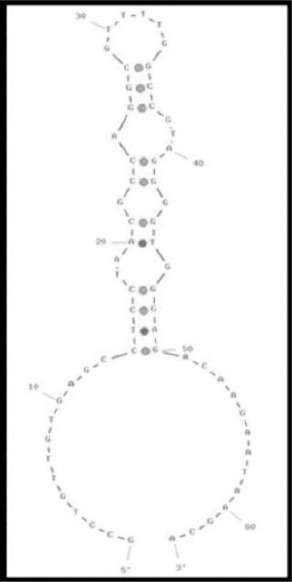
The secondary structure of aptamers K3 as determined by the Oligoanalyzer software v3.1.

Based on previous studies showing that stem-loop structures are important as binding regions of aptamers to targets[35,37], the secondary structure of the aptamer was studied. The predicted secondary structure of aptamer K3 is shown in Figure 5.

### Detection of CSF sample

**Fig. 6:**
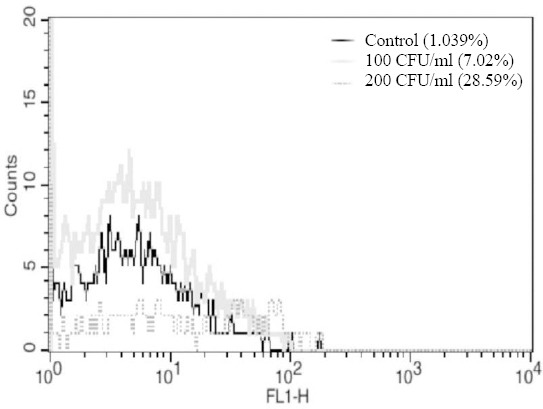
The fluorescent affinity of aptamer K3 to sterile CSF as the negative control (black curve), 200 CFU of *N. meningitidis* inoculated in CSF/ml (dark gray dotted), and 100 CFU of *N. meningitidis* inoculated in CSF/ml (light gray curve).

The known contaminated meningococcal CSF samples were detected by aptamer K3. The mean percent binding efficiency of aptamer K3 with two bacterial concentrations of 200 and 100 CFU ml^-1^ suspended in CSF samples were 28.59 and 7.02, respectively (Fig. 6). Aptamer K3 detected 10^2^ CFU of CSF isolated *N. meningitidis* prepared in binding buffer. These results were similar to those of *N. meningitidis* serogroup B inoculated in CSF from healthy individuals, whereas incubation of aptamer K3 with same concentration of *N. meningitidis* serogroup Y (ATCC 35561) in CSF did not show any binding affinity.

## DISCUSSION

Aptamers possess higher affinity to target on *N. meningitidis* cell surface and other bacterial pathogens like *Salmonella typhimurium*[18,19], *Campylobacter jejuni*[26], *Mycobacterium tuberculosis*[24], *Staphylo-coccus aureus*[23], *Streptococcus pyogenes*[27], *Shigella dysenteriae*[25], *Listeria* spp.[28], *Acinetobacter baumannii*[32], and *Listeria monocytogenes*[25]. Ligands other than aptamers such as antibodies or phage-binding proteins have been reported to be used for the capture of pathogens; they are not applied for non-cultivable or poorly cultivable pathogens[38]. In whole-cell SELEX approach, targets are presented in their native conformations which in turn increases the likelihood of the selected aptamers to be functional for their intended use. A greater affinity is displayed for viable cells than for inactivated cells by an aptamer selected using whole-cell SELEX[31,32,39]. Identification of the aptamer candidates is feasible by designing whole-cell SELEX with the addition of a label. As the addition of a label after the SELEX process can influence aptamer-binding affinity, we used a 5′ FITC ssDNA library for the aptamer selection procedure. This process resulted in the production of pre-labeled aptamers. In the whole-cell SELEX approach, flow cytometry was also used to sort cell-bound aptamers into pools based on fluorescence intensity. Aptamers which target more abundant cell binding sites are not necessarily those with tightest binding. Our findings are in agreement with those of Duan *et al*.[25], who produced ssDNA aptamers to *L. monocytogenes* in SELEX assay. Differentiation affinity between *N. meningitidis* serogroups was the core approach of the present study. Low to medium nM range of *K*_d_ values are usually found in aptamers employed in purified cell-surface moieties, while high nM to μM range of *K*_d_ values have previously been reported for whole cells specific aptamers[24-26]. In the present work, eight specific sequences were initially chosen, of which the aptamers K3 and K4 showed the highest activity (28.3 ± 8.9 pM and 39.1 ± 8.6 pM) resembling the previous reports[24,31,32]. Predicted K3 structure shows conserved large and stem-loop branches[18]. Moreover, the predicted secondary structure of DNA aptamer against *Shigella dysenteriae* indicates the location of two stem-loop branches on a larger central loop[25]. Consequently, both multiple hairpins and large end body loops can be key domains and potential binding sites. In this research, FIM ssDNA aptamers with binding specificity for *N. meningitidis* serogroup B were identified using a whole-cell SELEX approach. The selected aptamers other than K3 and K4 showed negligible binding affinity for other meningitis causing bacteria. Although in the whole-cell SELEX, aptamers are selected against live cells with no primary identity of their targets or molecular characteristics on the cell surface, comparing the affinity of K3 to serogroup B to that of other serogroups and related bacteria reveals that K3 is selected toward some strain-specific antigens of serogroup B cell surface.

As CSF contains various known and unknown physiochemical factors; therefore, we examined the binding affinity of the K3 with the meningococcal-positive patients’ CSF. In patients’ CSF, aptamer K3 showed 28.59 and 7% binding affinity at two bacterial concentrations of 200 and 100 CFU ml^-1^, respectively. Similar results were obtained with the sterile CSF contaminated with *N. meningitidis* serotype B. The findings indicated rational sensitivity and high specific binding affinity of aptamer K3 to *N. meningitidis*. This is the first report of its kind on the human CSF samples. These results together with those obtained from *N. meningitidis* suspended in binding buffer and in sterile CSF show bacterial dose dependence of the aptamer K3 sensitivity.

In conclusion, our results suggest that the use of a high-affinity K3 aptamer in a fluorescence-based quantification assay can be used as a rapid and simplified method for screening of *N. meningitidis* serogroup B in fabricated biological samples. It can also be used in an ELISA-based screening kit, if amplified with other labeled primers such as biotin.
